# Significant Biochemical, Biophysical and Metabolic Diversity in Circulating Human Cord Blood Reticulocytes

**DOI:** 10.1371/journal.pone.0076062

**Published:** 2013-10-08

**Authors:** Benoît Malleret, Fenggao Xu, Narla Mohandas, Rossarin Suwanarusk, Cindy Chu, Juliana A. Leite, Kayen Low, Claudia Turner, Kanlaya Sriprawat, Rou Zhang, Olivier Bertrand, Yves Colin, Fabio T. M. Costa, Choon Nam Ong, Mah Lee Ng, Chwee Teck Lim, Francois Nosten, Laurent Rénia, Bruce Russell

**Affiliations:** 1 Laboratory of Malaria Immunobiology, Singapore Immunology Network (SIgN), Agency for Science Technology and Research (A*STAR), Biopolis, Singapore; 2 Saw Swee Hock School of Public Health, National University of Singapore, Singapore; 3 Red Cell Physiology Laboratory, New York Blood Center, New York, New York, United States of America; 4 Shoklo Malaria Research Unit, Mae Sot, Thailand; 5 Departamento de Genética, Evolução e Bioagentes, Instituto de Biologia, Universidade Estadual de Campinas (UNICAMP), São Paulo, Brazil; 6 JEOL Asia Pte Ltd, Singapore; 7 Singapore-MIT Alliance, National University of Singapore, Singapore; 8 INSERM (Institut national de la santé et de la recherche médicale), UMR-S (Unité Mixte de Recherche) 665, Paris, France; 9 Institut National de la Transfusion Sanguine, Paris, France; 10 Universite Paris 7–Denis Diderot, Paris, France; 11 Department of Microbiology, Yong Loo Lin School of Medicine, National University of Singapore, National University Health System, Singapore; 12 Department of Bioengineering and Department of Mechanical Engineering, National University of Singapore, Singapore; 13 Mahidol-Oxford-University Research Unit, Bangkok, Thailand; 14 Centre for Tropical Medicine, University of Oxford, Churchill Hospital, Oxford, United Kingdom; Institut national de la santé et de la recherche médicale (INSERM), France

## Abstract

**Background:**

The transition from enucleated reticulocytes to mature normocytes is marked by substantial remodeling of the erythrocytic cytoplasm and membrane. Despite conspicuous changes, most studies describe the maturing reticulocyte as a homogenous erythropoietic cell type. While reticulocyte staging based on fluorescent RNA stains such as thiazole orange have been useful in a clinical setting; these ‘sub-vital’ stains may confound delicate studies on reticulocyte biology and may preclude their use in heamoparasite invasion studies.

**Design and Methods:**

Here we use highly purified populations of reticulocytes isolated from cord blood, sorted by flow cytometry into four sequential subpopulations based on transferrin receptor (CD71) expression: CD71high, CD71medium, CD71low and CD71negative. Each of these subgroups was phenotyped in terms of their, morphology, membrane antigens, biomechanical properties and metabolomic profile.

**Results:**

Superficially CD71high and CD71medium reticulocytes share a similar gross morphology (large and multilobular) when compared to the smaller, smooth and increasingly concave reticulocytes as seen in the in the CD71low and CD71negativesamples. However, between each of the four sample sets we observe significant decreases in shear modulus, cytoadhesive capacity, erythroid receptor expression (CD44, CD55, CD147, CD235R, and CD242) and metabolite concentrations. Interestingly increasing amounts of boric acid was found in the mature reticulocytes.

**Conclusions:**

Reticulocyte maturation is a dynamic and continuous process, confounding efforts to rigidly classify them. Certainly this study does not offer an alternative classification strategy; instead we used a nondestructive sampling method to examine key phenotypic changes of in reticulocytes. Our study emphasizes a need to focus greater attention on reticulocyte biology.

## Introduction

Reticulocytes are broadly defined as a group of immature erythrocytes which no longer contain a nucleus; however they are still in the process of undergoing significant membrane and cytoplasmic remodeling. It is this degenerate intracellular network (*substantia reticulo-filamentosa*), visualized using ‘sub-vital stains’ that is still the key diagnostic of the reticulocyte population. [1] While, reticulocytes only represent 0.6% to 2.9% of erythrocytes in adult blood and 1.7% to 5.0% in cord blood [2]; they are fundamental to the understanding of erythropoiesis, pathophysiology of various blood disorders including sickle cell disease and malaria and recently the *in vitro* production of red cells. [3].

Unfortunately and despite their importance; reticulocytes are often ‘lumped’ together as a biologically homogenous stage of red cell development, temporally located between the normoblast and normocyte stages. However, even a cursory glance of a basic reticulocyte preparation reveals a surprising level of heterogeneity in terms of the pattern and density of subvital staining. The obvious diversity within the reticulocyte population was first studied by Heilmeyer and Westhäuser [4] in the early 1930’s, where they classified four types of reticulocytes (group I, group II, group III and group IV) according to differential brilliant Crésyl blue staining patterns. Importantly, Heilmeyer and Westhäuser [4] carefully noted the relationship between the reduction in stained particles and the maturation of the reticulocytes. Despite this early work, few studies have seriously investigated the complex and fine scale changes evident in maturing reticulocytes. One major reason for this neglect is difficult and subjective process involved in sampling the small numbers of post-vitally stained reticulocytes by microscopy. Even if this task could be achieved; the RNA precipitating dyes and their solvents (i.e. Crésyl blue, new Methylene blue) will affect the accurate phenotyping of the reticulocytes, especially their biomechanical properties.

Since the 1950s, hematology has greatly benefited from the development of fluorescent stains and cytometry, providing a rapid and objective method to count reticulocytes.[5]– In particular, the use of Thiazole Orange (TO) [9] has proven to be of great utility in the clinical setting, also providing an alternative reticulocyte classification system known as the reticulocyte maturity index (RMI). [10], [11] The RMI, is derived from the higher TO staining regions (RMI = % moderate florescent (MFR)+% high fluorescent region (HFR)); though not explicitly stated the RMI corresponds to the total percentage of Heilmeyer groups I to III present in the blood. While TO staining (and the RMI) provides a useful diagnostic tool in a clinical setting, it has two important limitations when applied to developing detailed mechanistic understanding of reticulocyte maturation. First, the use nucleic acid intercalating florescent dyes such as TO and Ethidium may confound attempts to investigate staged reticulocyte ‘omics’ (i.e. Transcriptome). Second, as TO indiscriminately stains RNA and DNA; the presence of hemoparasites (i.e *Babesia* spp. and *Plasmodium* spp.) or pathological erythrocytic inclusions (i.e. Howell-Jolly bodies) may cause false positives. [12] ^,^ [13].

The discovery that the loss of transferrin receptor (CD71) provides a reliable marker for reticulocyte maturation perhaps represents the best chance at examining the characteristics of reticulocyte subsets with minimal artifact. [14] Kono *et al.* [15] utilized anti-CD71 antibodies and magnetic separation to examine the morphological characteristics of early (CD71 positive) versus mature (CD71 negative) human reticulocytes. This method yielded some important insights into reticulocyte detection, however did not provide differential information on the Heilmeyer subsets (groups I to III) that were clearly present in the CD71 positive fraction. [15] Additionally, the use of adult peripheral blood with its low proportion of reticulocytes (relative to cord blood) limits the yield of target material to phenotype; thus restricting the number of parallel experiments.

In this present study, we sorted highly enriched populations of cord blood reticulocytes by flow cytometry to obtain four distinct subsets of reticulocytes based on CD71 expression (CD71^high^, CD71^medium^, CD71^low^ and CD71^negative^).These high yielding cord blood samples have allowed us for the first time to accurately correlate morphological, biomechanical, metabolomic and immuno-phenotyping descriptions to the fine scale erythropoietic development of human reticulocytes.

## Materials and Methods

### Cord Blood Collection and Concentration

Cord blood samples were collected from obstetric clinics of the Shoklo Malaria Research Unit (SMRU), Mae Sot, Thailand; after written informed consent following ethical guidelines in the approved protocols; OXTREC 027-025 (University of Oxford, Centre for Clinical Vaccinology and Tropical Medicine, UK) and MUTM 2008-215 from Ethic committee of Faculty of Tropical Medicine, Mahidol University. Reticulocytes were isolated from cord blood as described previously. [16].

### Flow Cytometry Phenotyping and Sorting

Five hundred nanoliters of packed reticulocytes were stained with different uncoupled anti-human antibodies and with Fab’ anti-mouse Ab coupled with e660 (eBioscience®). The antibodies used in this study are described in Table S1. After these indirect staining, cells were directly stained anti-human CD71 FITC (Becton Dickinson®) to identify the different reticulocyte subsets based on CD71 expression (CD71^high^, CD71^medium^, CD71^low^ and CD71^negative^). Events’ acquisition was performed using a LSR II (Becton Dickinson) with subsequent data analysis using FlowJo software (Tree Star).

Reticulocyte subsets were isolated to >90% purity by flow cytometry using a FACS Aria II (Becton Dickinson) as described previously [17] but using anti-CD71 antibodies coupled to FITC (Becton Dickinson) and Hoechst (Sigma) staining to exclude leukocyte or undiagnosed hemoparasite contamination.

### Optical and Electron Microscopy

**Figure 1:**
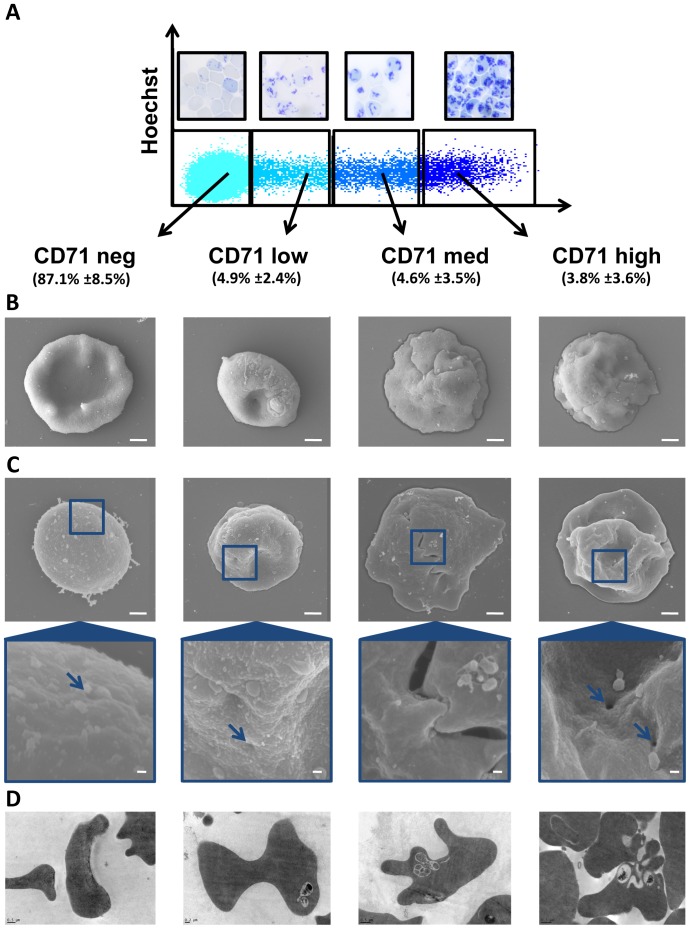
Flow cytometry gating strategy and representative morphology of reticulocyte samples. (**A**) Flow cytometry dot plot showing CD71 and Hoechst staining using to define strategy gating for different reticulocyte subset sampling: CD71^high^, CD71^medium^, CD71^low^ and CD71^negative^. The mean percentage of each subsets are inserted in brackets (n = 14). Cord blood reticulocyte subsets stained with new Methylene blue (see insets). (**B**) Morphology of cord blood reticulocytes was observed by scanning electron microscopy. The scale bars represent 1 µm. (**C**) The blue bordered insets show key nanostructures on the surface of the reticulocytes. The scale bars in these insets represent 100 nm. (**D**) Intracellular reticulocyte structures were observed by transmission electron microscopy.

Microscopic examination (100×oil immersion objective) of reticulocytes was performed after sub-vital staining with new methylene blue (Sigma-Aldrich®). For Scanning Electron Microscopy (SEM) and Transmission Electron Microscopy (TEM) imaging, standard protocols were used. It is important to note that SEM and TEM were imaged from a FACs sorted population (purity >85%) of between 20,000 and 50,000 cells so the reticulocyte density on the coverslip is very low. Consequently we do not provide low power images where you would see few of the important features we wish to discuss, additionally cells in the CD71 high sorts have a “charge effect”(common to all medium and high CD71 samples in all the CB isolates we examined). This charge effect is not very pleasing to the image and obscures many of the cells (appearing as fuzzy horizontal lines on some of the nascent reticulocytes) this along with the large amount of blank space between the cells lead us to select only a couple of representative cells from our scans, at high magnification (Fig. 1). However to satisfy the need for more samples and to demonstrate we did not just “cherry pick” an unusual reticulocyte image; we provide a Figure S1 showing 8 images for each sample set.

### Micropipette Aspiration

The shear modulus of the various reticulocytes was based on the micropipette method adapted from LaCelle *et al.* [18], Leblond *et al.* [19] and Chasis *et al.* [20] using a hemispherical cap model. Single cells were picked up and aspirated at a pressure drop rate of 1 pa/s ImageJ Software was used to measure the aspirated length of the RBC membrane.

### Cytoadherence

We assessed the ability of sorted cord blood reticulocytes subsets (CD71^high^, CD71^medium^, CD71^low^, CD71^negative^) to adhere to platelets or Chinese hamster ovary cells (CHO and CHO-ICAM-1) performing static cytoadhesion assays as described elsewhere [21].

### Metabolomics

One million sorted cord blood reticulocytes from each sample gates were freeze-dried and treated with ice-cold methanol spiked with FMOC-glycine as internal standard. The GC/MS analysis was carried out as per Xu *et al.* [22] The selected marker compounds from GC/MS analysis were identified by comparison of mass spectra and retention time with those of reference standards, and those available in libraries (NIST 2005).

### Statistical Analysis

Except when indicated six cord blood isolates were used for each experiment (n = 6). As all of the comparative observations were made on subsets derived from a cord blood isolate, analyses must be considered as repeated measures. As this data was nonparametric, comparisons utilized Friedman tests with a Dunns Post hoc analysis test for pair wise comparisons. All statistical analysis used Prism 5 for Windows (version 5.01), Software MackievTM.

## Results

### Morphology of Human Cord Blood Reticulocytes

Reticulocyte samples were obtained from cord blood as previously described by Russell et al. providing upto193 µl of packed cells. [16] After labeling with a fluorescent anti-CD71 antibody, reticulocyte fractions were separated by flow cytometry sorting based on CD71 (transferrin receptor) expression. Most of the reticulocytes were CD71^negative^ (87.1% ±8.5%), with the remaining CD71 positive population split into three equal reticulocyte subsets; CD71^low^ (4.9% ±2.4%), CD71^medium^ (4.6% ±3.5%) and CD71^high^ (3.8% ±3.6%) (Fig. 1A). The CD71^high^ and CD71^medium^ populations have a macroscopic morphological similarity with R1 reticulocyte population described previously [23], and CD71^low^ and CD71^negative^ populations to R2 reticulocyte population. Post-vital staining each of these sorted subgroups with new Methylene blue, revealed a network of precipitated ribosome material, the density of which decreased relative to CD71 expression. (Fig. 1A) as described previously. [15] But it is very important to note that post-vital staining of CD71 positive subset with new Methylene blue, revealed a majority of group II and group III of Heilmeyer’s classification. [4].

The surface morphology of the sorted reticulocyte stages was then observed by SEM. The most immature circulating reticulocytes (CD71^high^ and CD71^medium^) showed characteristic cytoplasmic retractions associated with deep membrane sutures (Fig. 1C). Interestingly most of the CD71^high^ and CD71^medium^ reticulocytes have striking ‘zipper -like’ sutures, rarely seen on CD71^low^ and never seen on CD71^negative^ reticulocytes (Fig. 1C). The morphology CD71^medium^ and CD71^low^ subsets showed a progressive reorganization of the membrane and cytoplasm, characterized by the initiation of concavity (Figs 1B and 1C). The most mature reticulocyte subset (CD71^negative^) morphology clearly showed the development of a proto-biconcave shape. The membrane surface the CD71^high^, CD71^medium^ and CD71^low^ subsets are covered with pit like structures ∼100 nm in diameter. These pits are rarely observed in CD71^negative^ samples (Fig. 1C).

The intracellular structures of reticulocytes were imaged using TEM (Fig. 1D). CD71^high^ subsets showed a tormented aspect with numerous membrane convolutions. Large multivesicular elements containing round bodies appeared clearly inside cytoplasm. The multivesicular elements were absent from cytoplasm of CD71^negative^ reticulocytes. The characteristic biconcave shape is clearly visible on two illustrated sagittal cuts CD71^negative^ subset (Fig. 1D).

### Immuno-phenotyping of Reticulocyte Subsets in Human Cord Blood

**Figure 2:**
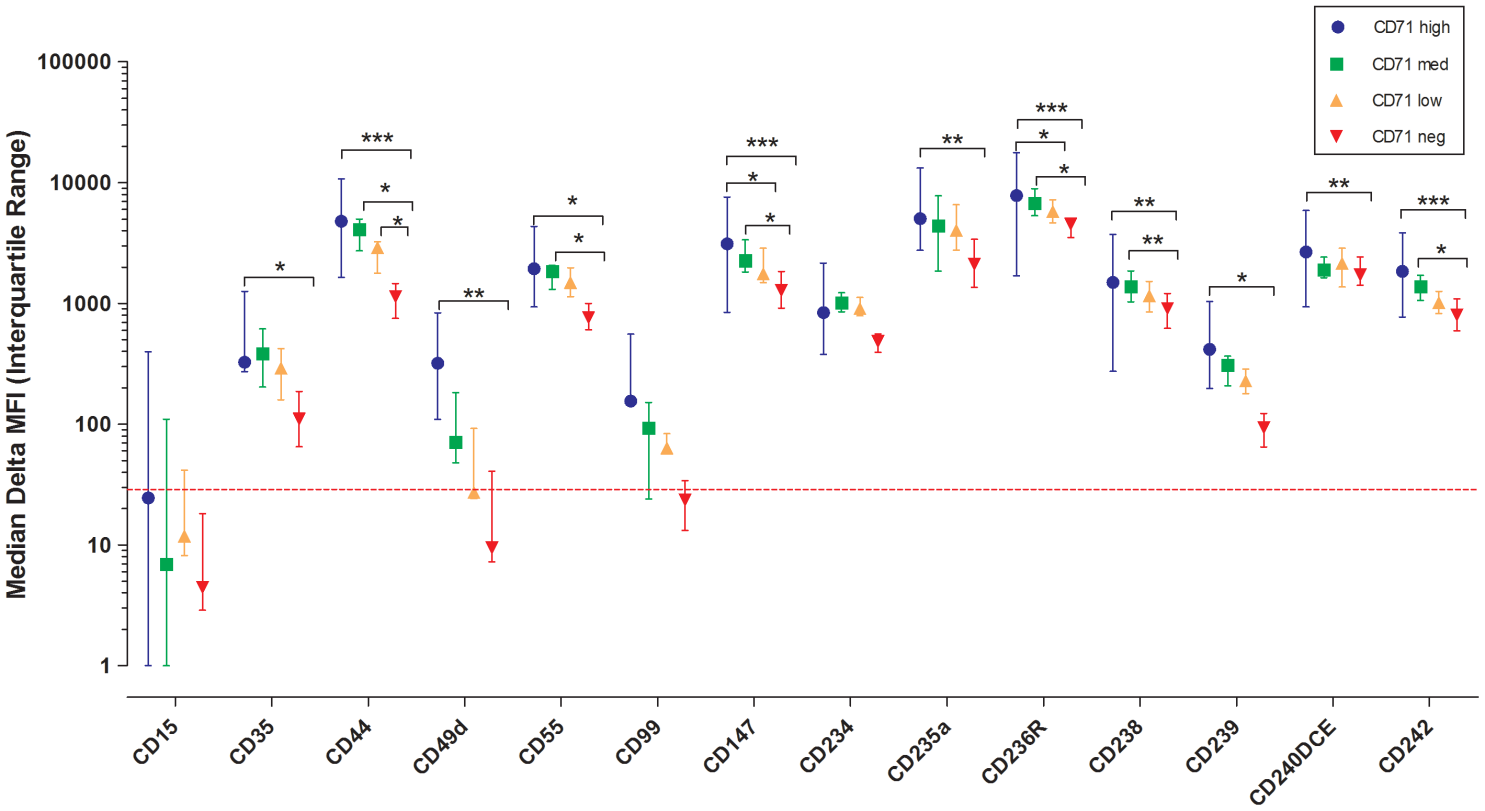
Immunophenotyping of reticulocyte samples. Difference of between mean fluorescence intensity with different erythrocytic antigens and mean fluorescence intensity with secondary antibody on reticulocytes sampled from the four CD71 gates (CD71^high^, CD71^medium^, CD71^low^ and CD71^negative^) (Delta of Mean Fluorescence Intensity (MFI)). The dotted red line represents the threshold of positivity based on the CD15 staining.

We next analyzed surface cell marker expressions of reticulocytes by flow cytometry (Fig. 2 and Figure S2). Reticulocyte suspensions were pre-stained with anti-CD71 and all markers were counter-stained using secondary anti-mouse coupled e660 antibody. Table S1 provides the list of different markers for reticulocyte immuno-phenotyping. As expected, Lewis-X (CD15) antigen expression was completely absent on all reticulocyte subgroups. [24] The expression of CD44 (Hermes), CD147 (Basigin), CD236R (GPC) and CD242 (ICAM-4) was significantly decreased between CD71^high^ and CD71^negative^ reticulocyte subgroups (P<0.001) (Fig. 2). In all these cases, post hoc analysis showed a significant reduction between CD71^High^ and CD71^Low^; and CD71^Medium^ and CD71^Negative^ (Fig. 2). The decrease of expression was relatively less for CD49d (alpha 4-Integrin), CD234 (Duffy), CD235a (GPA), CD238 (Kell), CD240DCE (Rhesus 30) (P<0.01) and even less for CD35 (CR1) CD55 (DAF) CD239 (Lutheran), however still statistically significant (P<0.05). While the expression of CD99 (MIC2) tended to decrease, this did not reach significance due to an already low CD99 signal on the CD71^high^ subgroup and a high auto-fluorescence background affecting the signal detection.

We wish to emphasize that that we present the Delta of Mean Fluorescence Intensity (MFI) (between stained cells and those incubated only with the secondary anti-body for each CD71 categories)(Fig. 2) to negate the effect of confounders such as reticulocyte size and/or autofluorescence.

### Biomechanical Properties

**Figure 3:**
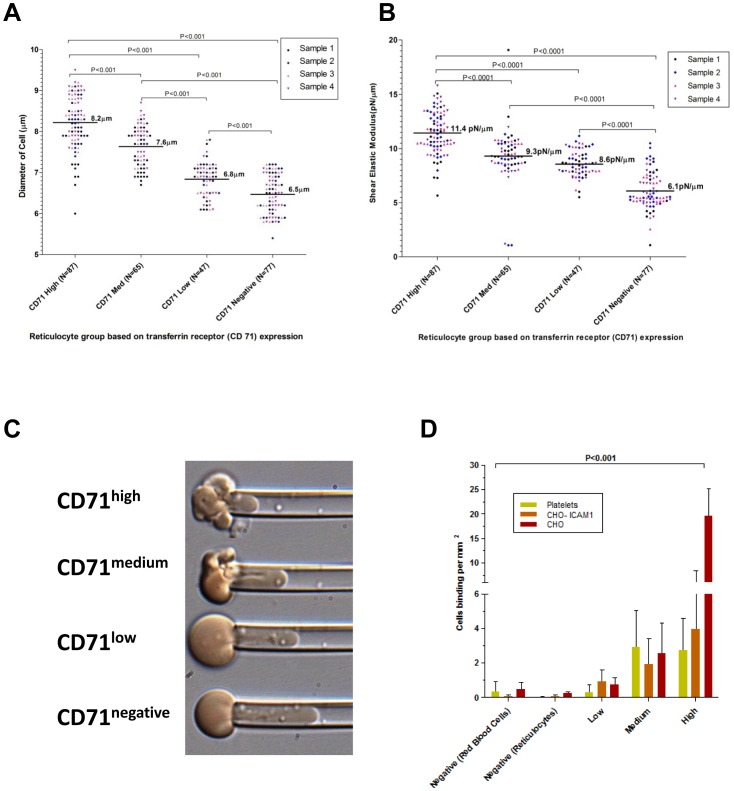
Biomechanical properties of reticulocyte samples. The size and biomechanical characteristics of CD71 sorted cord blood reticulocytes (different color markers represent each of the 4 isolates used). (**A**) The median diameter (um−/+IQR) of live reticulocytes in physiological media. (**B**) The median shear flow modulus (−/+IQR) (high shear flow modulus indicates reduced membrane deformability) of the reticulocyte membranes as measured by micropipette aspiration. (**C**) Representative micropipette aspiration images of CD71^high^, CD71^medium^, CD71^low^ and CD71^negative^ (top panel to bottom respectively) measured at an identical pressure. (**D**) The relative cytoadhesiveness of different reticulocyte samples on CHO cells (two types) and activated platelets.

We next investigated the biomechanical properties (size, deformability and adhesiveness) critical for the *in vivo* circulation of reticulocytes. The average size of different reticulocyte subsets was measured by optical microscopy using fresh cells from four different donors. CD71^high^ reticulocytes subset had on average, a diameter of 8.2 µm (n = 87 cells), CD71^medium^ subset of 7.6 µm (n = 65 cells), CD71^low^ subset of 6.8 µm (n = 47 cells) and CD71^negative^ subset of 6.5 µm (n = 77 cells) (Fig. 3A). The size cell decrease was significant between each groups (p<0.001).

Micropipette aspiration for shear elastic modulus utilized the same four cord blood samples as used in the size determination. The median shear elastic modulus was11.4 pN/µm (n = 87 cells), 9.3 pN/µm (n = 65 cells), 8.6 pN/µm (n = 47 cells), 6.1 pN/µm (n = 77 cells) for CD71^high^, CD71^medium^, CD71^low^ and CD71^negative^ subsets respectively (Fig. 3B). The decrease of shear elastic modulus values with increasing reticulocyte stage maturity reflected an increase in deformability (P<0.0001). The significant increases in reticulocyte deformability clearly reflected by the increasing length of the aspirated reticulocyte in the micropipette (Fig. 3 C).

A semi static adhesion assay was used to measure the relative cytoadhesiveness of reticulocyte subsets to platelets, CHO-ICAM-1 cells and CHO cells. Immature reticulocytes (CD71^high^ and CD71^medium^) are significantly more cytoadhesive compared to the more mature reticulocyte subsets (P<0.001) (Fig. 3D).

### Reticulocyte Metabolome

**Figure 4:**
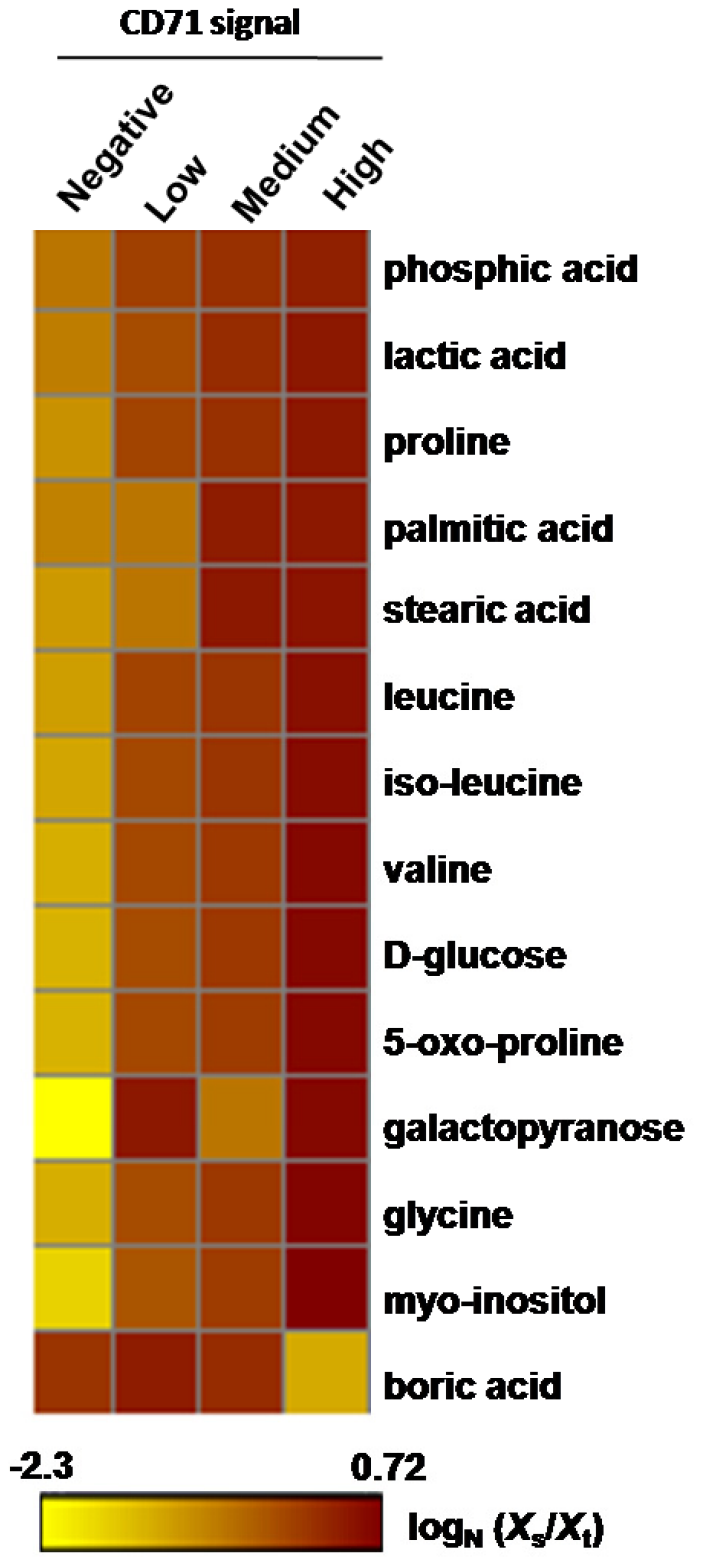
Metabolomic profile of reticulocyte samples. Heat map of 14 metabolomic markers (GC-MS) found in differential amounts (dark brown indicating a greater quantity) in CD71 high, medium, low and negative reticulocyte populations. Boric acid was the only marker that increased as the reticulocyte matured from CD71 high to negative. The normalized level of marker scale is shown at the bottom of the heat map.

Gas chromatography–mass spectrometry analysis of the cord blood subsets revealed 19 metabolites with a signal sufficient to provide a profile of their relative levels (Table S2). However only fourteen provided a complete set of data for each reticulocyte subset (Fig. 4). These markers cover a wide range of metabolic pathways and include amino acids, nucleotides, metabolic intermediates and sugars. Except for boric acid all the markers were attenuated as the reticulocyte matured.

## Discussion

**Figure 5:**
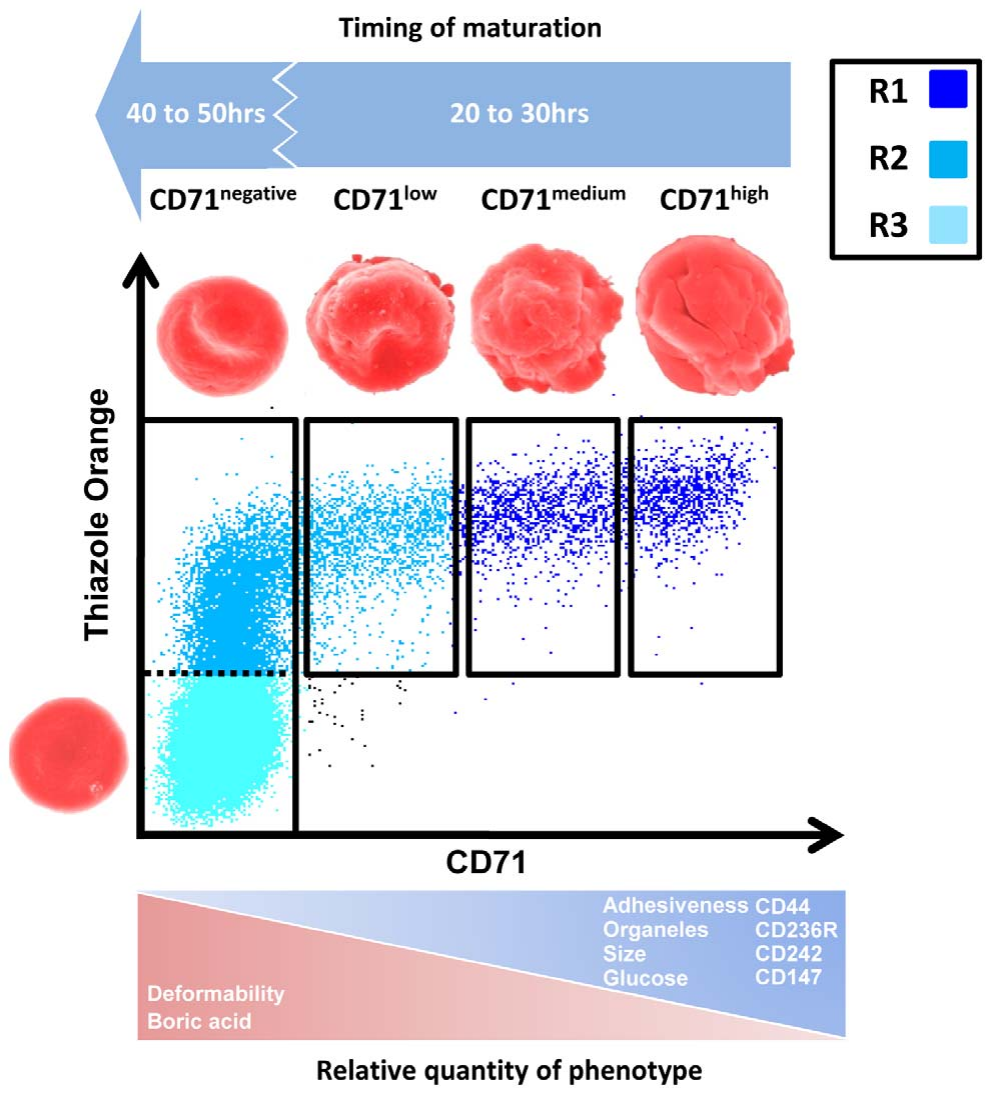
Summary of the key phenotypic changes in maturing human cord blood samples. An overview of the key biochemical, biophysical and metabolic changes occurring in the subsets of circulating cord blood reticulocytes relative to RNA content as measured by thiazole orange. Our finding are also put into the context of the ‘R’ reticulocyte characterization (R1 = Immature reticulocytes, R2 = Mature reticulocytes and R3 = Normocytes). [23].

Sampling of CD71 labeled erythrocytes from *ex vivo* cord blood reveals a previously unreported level of phenotypic diversity amongst maturing reticulocytes. It must be stressed that the four sampling divisions used in this study do not constitute a new classification system. Instead, we used these sequential sorting gates to provide improved resolution of sampling along the continuum of reticulocyte maturation; using a labeling method that minimizes confounding effects to measurements of the various phenotypes. At a biological level, one should be cautious about any classification scheme that lumps together heterogeneous reticulocytes into super groups (i.e. R1 and R2) [23] or CD71 positive and negative or high-, medium-, low- fluorescent ratio region (HFR, MFR, and LFR [15]; however there is a clinical imperative for diagnostic labels that indicate a patient sample has an increased proportion of reticulocyte stages. To this end we designed a discussion figure (Fig. 5) that provides a synteny between the CD71 expression levels and the standard methods used for reticulocyte classification. This figure not only aids in familiarizing our sampling strategy with important classification schemes, but also summarizes our key findings.

Although many earlier studies have examined the morphology of human reticulocytes with electron microscopy, [25] our study utilizing flow cytometry sorting, is the first to directly link R1 and R2 populations with a specific SEM and TEM morphology. Certainly, the gross morphological restructuring of the reticulocytes as they mature provides the most compelling evidence for reticulocyte heterogeneity. While the ‘wrinkled globular’ shape of reticulocytes from the CD71^high^ and CD71^medium^ samples share many morphological similarities, they bear almost no resemblance to the proto-biconcave and biconcave shapes found in the reticulocytes sampled from the CD71 ^negative^ gate. As biconcave-like geometries confer a greater deformability, the loss of the globular shape first detected in the CD71^low^ reticulocytes undoubtedly contribute to its decreased stiffness. In addition to shape effect, work by Chassis *et al* in 1989 on *ex vivo* matured reticulocytes also shows significant increases to deformability and also mechanical stability in later stages of reticulocyte development. [20] Chassis *et al.* [20] also speculates on the contribution of membrane skeleton remodeling to reticulocyte deformability, especially the assembly of spectrin and the 4.1R complex. It is hoped that future studies specifically examine how the development of cytoskeleton in these reticulocyte stages contribute to their biomechanical changes.

The recent study by Griffiths *et al* [26] shows the morphology of R1 *in vitro* matured reticulocytes, which are clearly different to those observed in our *ex vivo* human cord blood samples. For example, the ‘R1’ reticulocyte presented by Griffiths et al [26] shows none of the deep ‘zipper-like’ sutures characteristically observed in the early stage reticulocytes sampled from out CD71^high^ and CD71^medium^ gates (which correspond to the R1 stages [23]).

In addition to major changes in shape, the size of the reticulocytes also significantly decreases. [27] While our study measured unstained reticulocytes in a wet preparation, smears of post-vitally stained reticulocytes also show a noticeable reduction in cellular diameter. It is a bit surprising that Heilmeyer neglected to reflect the obvious change in reticulocyte size in the figure he used illustrate his classification system. [4].

At the nanoscale, 80 to 120 nM diameter ‘pit structures’ are readily observable on all reticulocytes, except those sampled from the CD71^negative^ gate. These ‘pit structures’, reported by many other groups, are clathrin pits, containing the CD71 receptor (transferrin receptor) needed for the import of iron into the developing erythrocyte. [28], [29] (Blue arrows Fig. 1C). It is logical to assume that the lack of observable pits in CD71 ^negative^ gated reticulocytes indicates hemoglobin synthesis capacity of these mature reticulocytes is virtually nonexistent. [30], [31]. Aside from pits, the other nanofeatures present on the earlier stages of reticulocytes (CD71^high^ and CD71^medium^) are ∼100 nM bubble-like excrescences. These exosomes are involved in the expulsion of CD71 and other membrane associated proteins such as CD44, CD147 and β1 integrin. [26] ^,^ [32].

One key set of data revealed in this study is the fine scale immune-phenotyping of the various reticulocyte stages. While other studies note the expression of different erythroid specific markers on *in vitro* matured reticulocytes, [32] ^,^ [33] this is the first study to attribute the expression of erythroid-specific markers within the spectrum of reticulocyte development. While the maturation of reticulocytes is marked by a general reduction in the expression of markers; this reduction is particularly significant (P<0.001) in the case of CD44, CD147, CD236R, and CD242. Of particular note, our results show a sizable drop in CD44 expression, concurring with the findings of Chen *et al.* who showed a similar sharp drop in CD44 during the maturation of mouse reticulocytes. [32]. It should be borne in mind that this study utilized cord blood reticulocytes and one would expect to find some differences between our samples and those concentrated from adult donors (for example CD15 was completely absent from cord blood reticulocytes, this being noted also by Lewis *et al.* [24]).

Our immunophenotypic data lends support to the use of rodent models in the study of reticulocyte biology. First of all, reticulocyte maturation is concomitant with decreasing CD71 expression [34]. Furthermore, our data showing a significant reduction in CD242(ICAM-4) expression (Fig. 2) links with observations in the murine model where the level of CD242 expression has a crucial role in erythroblastic island formation [35] and the removal of senescent mouse RBCs in the spleen. [36] ^,^ [37] Additionally, a rodent model developed by Khandelwal *et al.* [38] suggested the initial sharp drop in CD147 expression, is associated with reticulocyte maturation, as we also found in this study. However, CD147 was still highly expressed in normocytes and plays also a crucial role in splenic clearance of senescent erythrocytes. [39].

It should be borne in mind that significant changes in the expression of erythroid markers such as CD242 and CD236R during reticulocyte maturation will result in alterations to the stability of the membrane skeleton. This is because CD242 (with CD235a) and CD236R (with CD44, CD147, CD238 and CD240DCE) are linked with the Band 3 complex and 4.1R complex respectively. [40], [41].

The general decrease in erythroid transmembrane receptors clearly affects overall cytoadhesiveness of reticulocytes which also decreases substantially as they mature. It should be noted that our observation that reticulocytes are relatively ‘sticky’, is not new, with Key *et al.* making particular note of reticulocyte adhesion over 80 years ago. [42] The decrease in this adhesiveness is essential at the normoblast stage to allow reticulocyte egress through sinus wall to the peripheral blood stream. [43] The process of reticulocyte egress in the mouse model is controlled by erythropoietin (EPO) which modulates the expression of VCAM-1 ligands as CD49d. [44] Certainly our study shows an almost complete disappearance of CD49d on reticulocytes sampled from the CD71^negative^ gate.

Our results on the metabolomics of reticulocytes while somewhat preliminary, the data clearly shows a general attenuation of important metabolic markers especially that of glucose which are consumed throughout the maturation of the reticulocytes. In perhaps the best study on the biochemical basis of reticulocyte maturation, [45] it was noted a differential rates of glucose consumption due to the fact that polynucleotide removal and reticulocyte maturation is glucose dependent. Of note Boric acid is the only marker we observed increasing in the more mature erythrocytes. It is a little known fact that mammalian cells have a borate transporter (NaBC1 or SLC4A11) homologous to the AtBor1 boron transporter found in plants. [46] While we can only speculate about the presence of NaBC1 transporters in human red blood cells, [47] only SLC4A1 (Band 3) in SLC4 family transporter protein was described at the surface of RBC [48]s. Boron homeostasis has not been studied in mammalian cells and is outside the scope of this study; however it is known that boron interacts with organic compounds containing hydroxyl groups to form boric acid and borate (Park et al 2004). It is interesting to note that the antioxidant properties of small amounts of this weak acid such as Boric acid, may be of advantage to protecting the fully matured normocyte. [49].

One obvious omission in this study is the development time between each of the reticulocyte sub stages. In a preliminary set of experiments, we found the total time from the immature reticulocytes (seen predominantly in the CD71^high^ gate) to mature normocytes was ∼140 hrs under *ex vivo* conditions, a maturation time almost two fold higher than the generally excepted 80 hrs. [50] However, we need to interpret this data with caution since reticulocyte maturation under *ex vivo* conditions bears little resemblance to the *in vivo* conditions. In the excellent review published in 1953 [50] on reticulocyte maturation, most in vivo studies show that the development time from Heilmeyer stage I to III is relatively short (∼24 hrs) compared to the maturation time of Heilmeyer stage IV to normocyte (∼56 hrs) (Fig. 5). Indeed, no more than ∼20% of the reticulocytes found in our cord blood preparations were CD71 positive (Heilmeyer Stages I, II, III), supporting the idea that these stages are short-lived compared to Heilmeyer stage IV reticulocytes.

Clearly our results demonstrate the significant heterogeneity in the reticulocyte population. However while studies continue to treat reticulocytes as a population average, the biological consequences of phenotypic differences (i.e. biomechanics and receptor expression) in reticulocyte subgroups will remain unknown. A case in point is the sole transcriptome study on reticulocytes, [51] that would have yielded even more insights into this important stage of hematopoiesis if mRNA was extracted from sequential reticulocytes samples, rather than the whole population. In future, such a study will benefit from the development of high throughput cell sorting (20,000 events per second with Influx BD flow cytometer) that will provide sufficient material for such fine scale ‘omic’ studies on reticulocyte sub-populations. While this present study was limited to cord blood reticulocytes, future studies should also investigate adult reticulocyte populations. Even though we make reference to specific stages of reticulocyte development, we fully acknowledge these are somewhat imperfect labels to describe what is in fact; a dynamic and continuous maturation process. It must be stressed that the recommendations of our study relate to hematological research on concentrated reticulocyte preparations; not clinical reticulocyte enumeration on whole blood samples. The CD71 expression sorting method utilized in this study is not appropriate as a diagnostic tool, when compared to the superior protocols reviewed by Riley *et al.* [13]. However; we are confident that reticulocytes sampled in the manner we described, will provide useful when examining the preferential targeting of reticulocytes of a particular maturity, by parasites such as *Plasmodium vivax*.

We hope this study reinvigorates research hematologists to take a closer look at reticulocyte subsets, so that future studies accurately reflect their true heterogeneity and specific association with human biology and pathophysiology.

## Supporting Information

Figure S1 Morphology of CD71^high^, CD71^medium^, CD71^low^ and CD71^negative^ reticulocytes visualized by Scanning Electron Microscopy. The scale bars represent 1 µm.
(TIF)

Figure S2 Flow cytometric phenotyping of reticulocyte sampling sets. For each staining the different reticulocyte samples are defined with CD71 FITC staining (CD71^high^, CD71^medium^, CD71^low^ and CD71^negative^). Each erythrocytic antigen staining (blue histogram) are compared with secondary antibody staining (grey histogram).
(TIF)

Table S1 List of Antibodies used in the study. Cluster of differentiation(CD) number, alternative names, function at the surface of RBCs, clone and manufacturers are detailed for each antibody.
(TIF)

Table S2 GC-MS data on 19 metabolomic markers found in differential amounts in CD71 ^high, medium, low and negative^ reticulocyte population samples. Five of these did not produce values for CD71 ^negative^ reticulocytes (gray lines) and this data was not included on the heat map.
(TIF)
