# Supplementary figures and images for: Significant Biochemical, Biophysical and Metabolic Diversity in Circulating Human Cord Blood Reticulocytes
Source: PLoS One. 2013 Oct 8;8(10):e76062. doi: 10.1371/journal.pone.0076062 (PMC3793000; doi:10.1371/journal.pone.0076062)

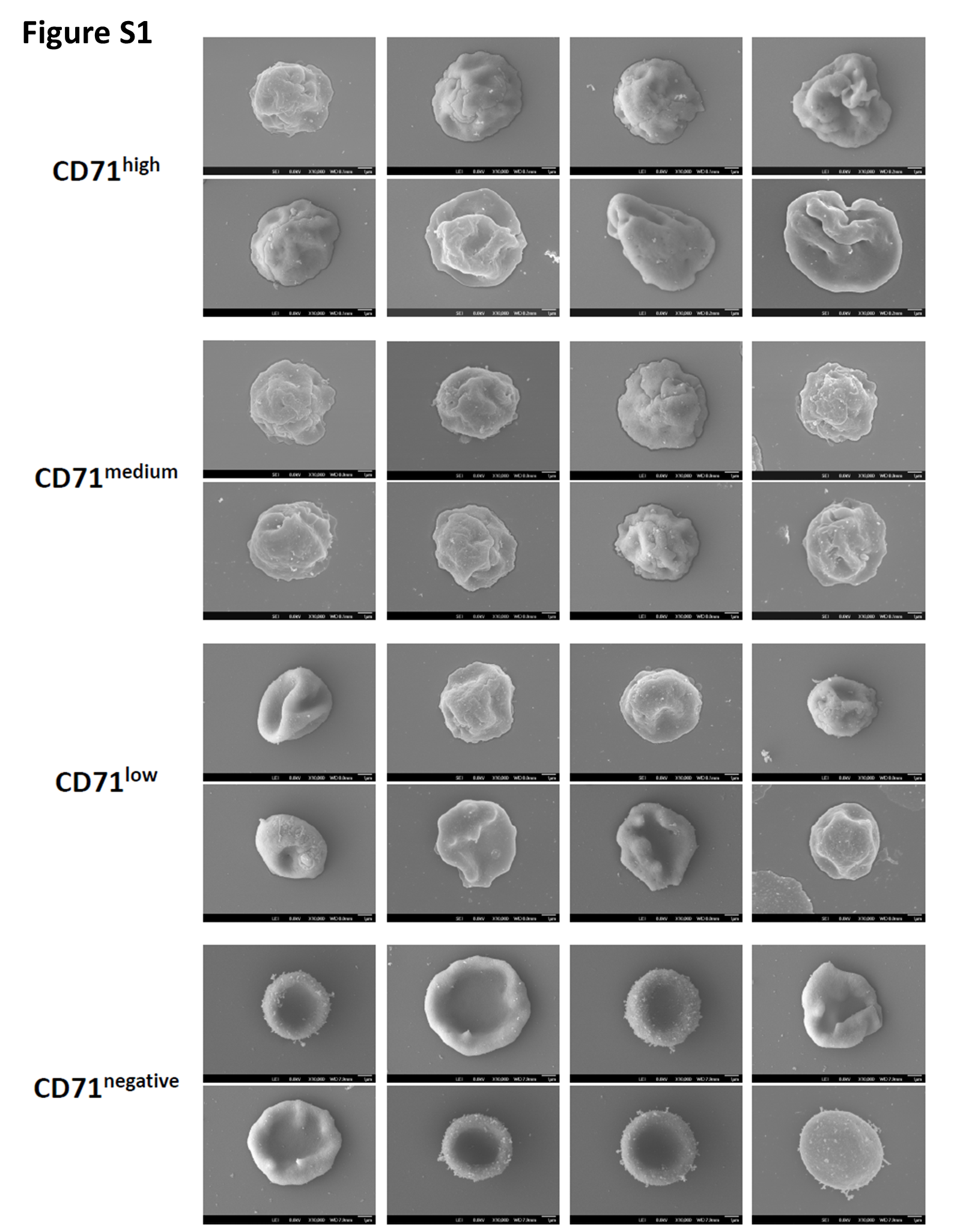

Supplement: Figure S1 — Morphology of CD71high, CD71medium, CD71low and CD71negative reticulocytes visualized by Scanning Electron Microscopy. The scale bars represent 1 µm. (TIF) [file pone.0076062.s001.tif]

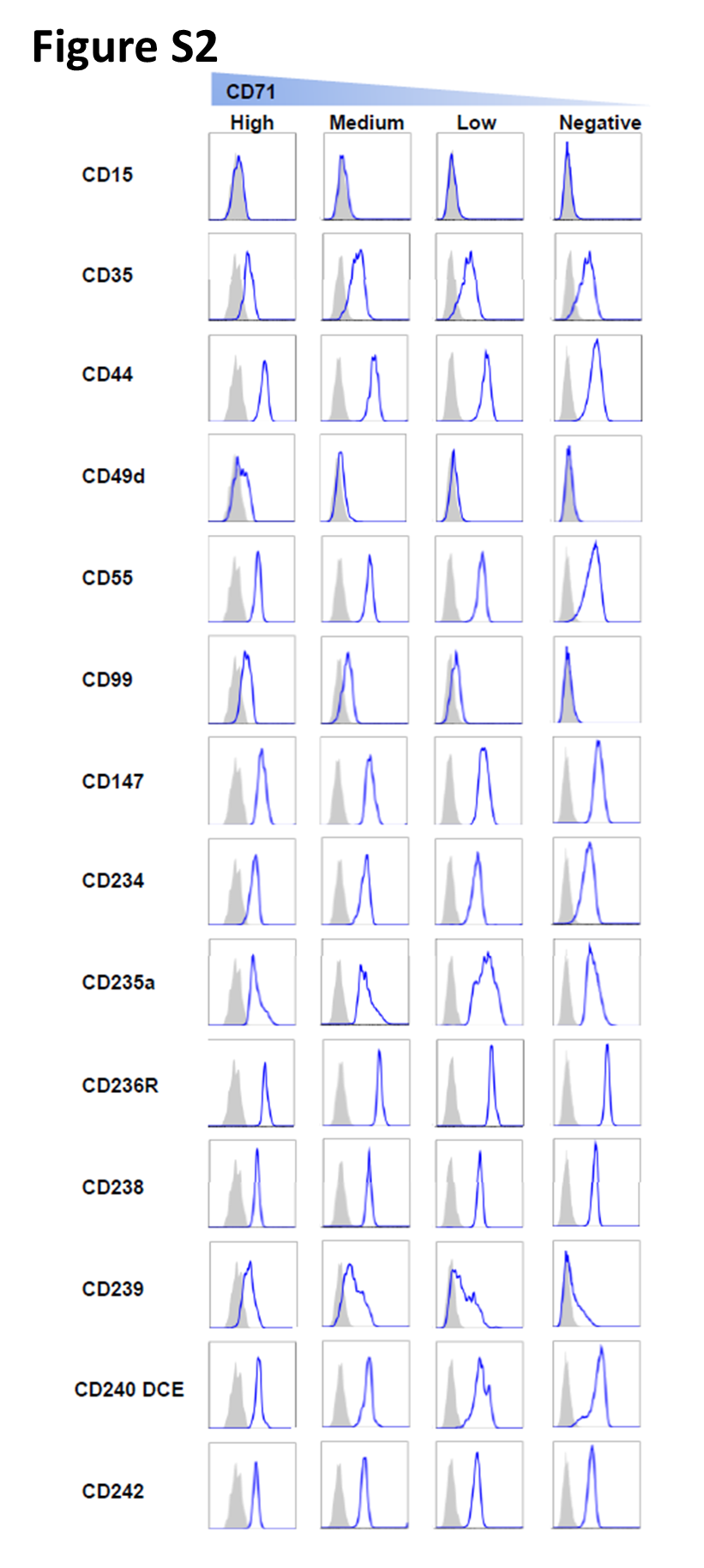

Supplement: Figure S2 — Flow cytometric phenotyping of reticulocyte sampling sets. For each staining the different reticulocyte samples are defined with CD71 FITC staining (CD71high, CD71medium, CD71low and CD71negative). Each erythrocytic antigen staining (blue histogram) are compared with secondary antibody staining (grey histogram). (TIF) [file pone.0076062.s002.tif]

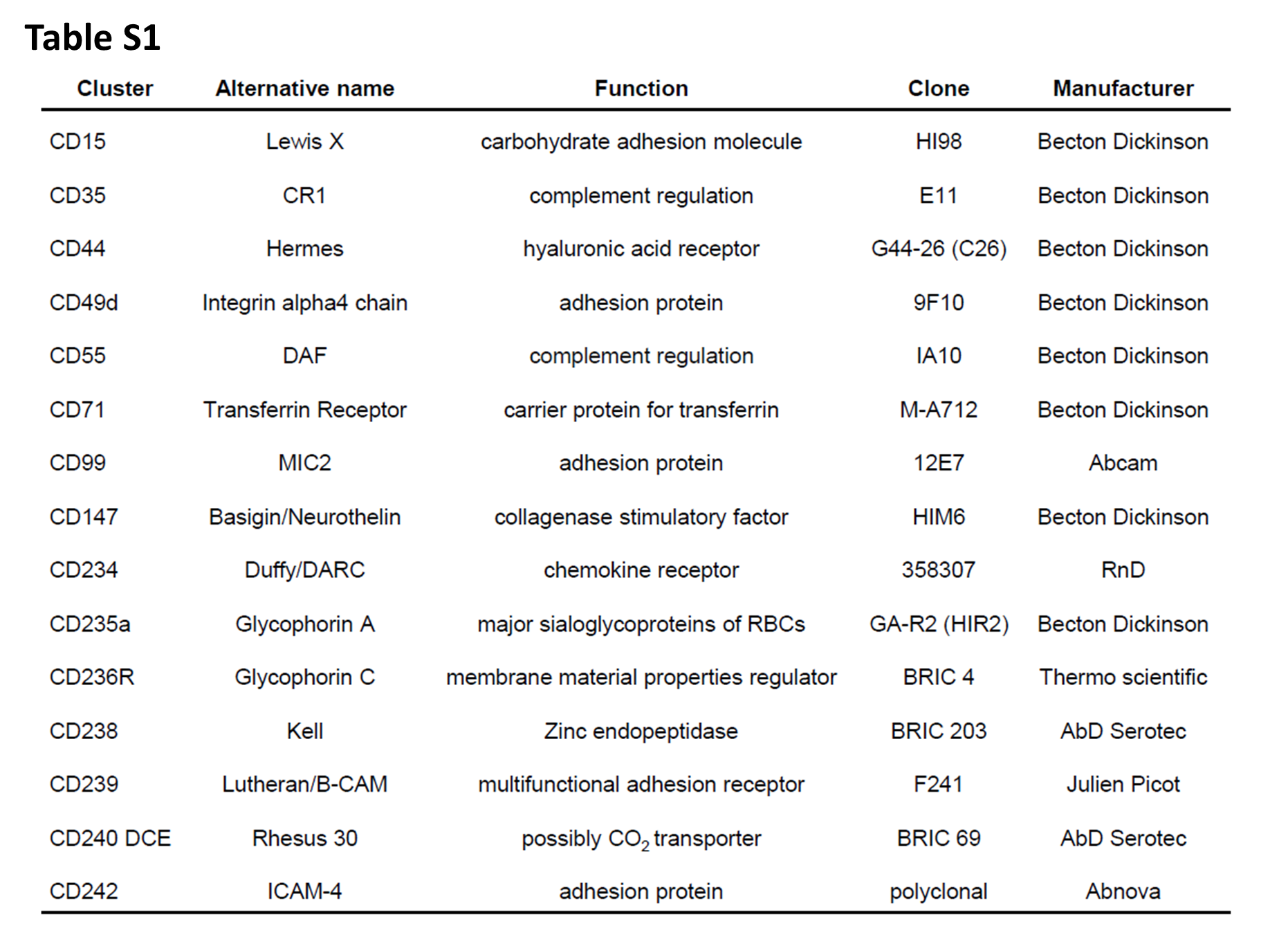

Supplement: Table S1 — List of Antibodies used in the study. Cluster of differentiation(CD) number, alternative names, function at the surface of RBCs, clone and manufacturers are detailed for each antibody. (TIF) [file pone.0076062.s003.tif]

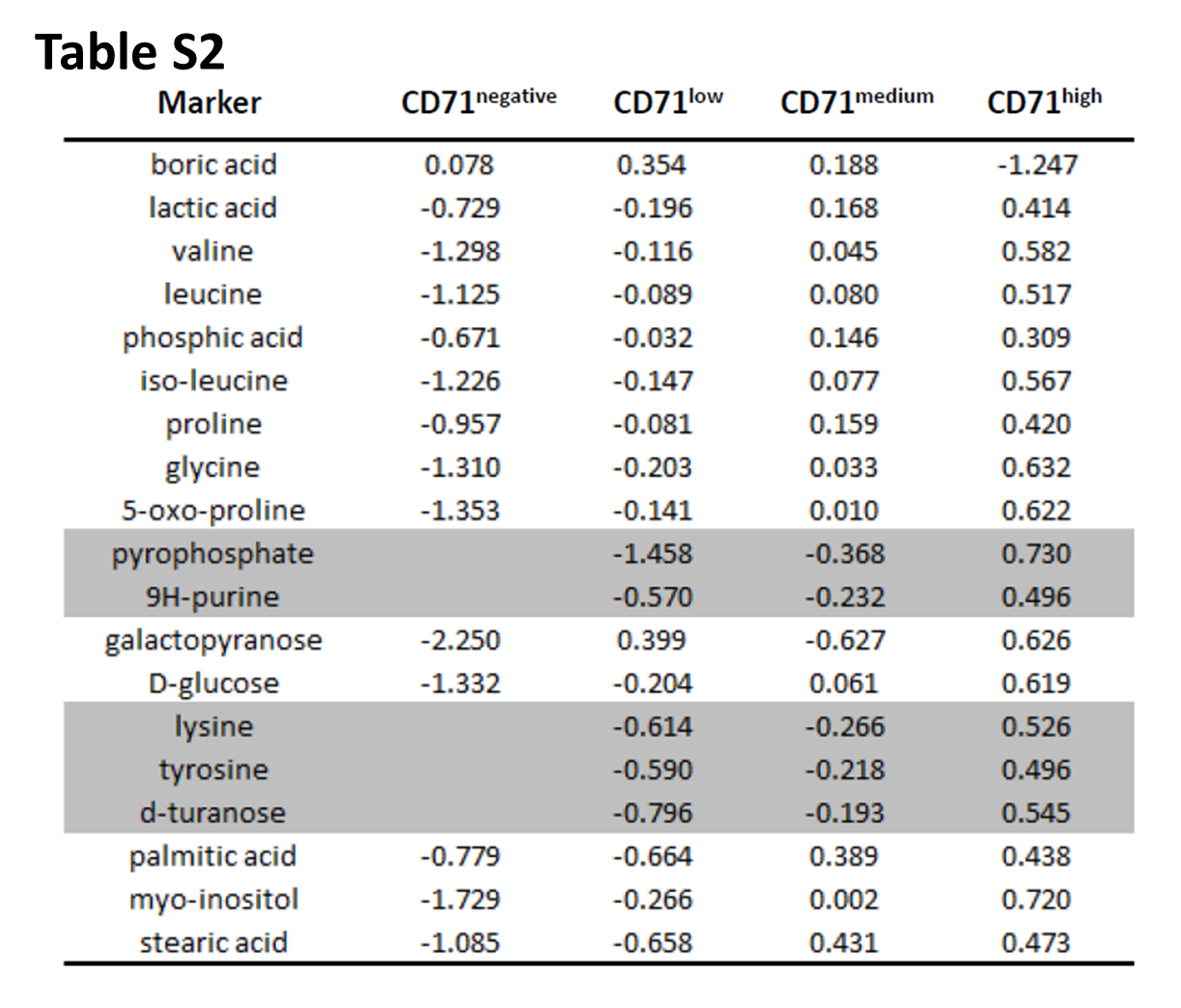

Supplement: Table S2 — GC-MS data on 19 metabolomic markers found in differential amounts in CD71 high, medium, low and negative reticulocyte population samples. Five of these did not produce values for CD71 negative reticulocytes (gray lines) and this data was not included on the heat map. (TIF) [file pone.0076062.s004.tif]
